# Development of Sustainable, Mechanically Strong, and Self-Healing Bio-Thermoplastic Elastomers Reinforced with Alginates

**DOI:** 10.3390/polym14214607

**Published:** 2022-10-30

**Authors:** Saul Utrera-Barrios, Ornella Ricciardi, Sergio González, Raquel Verdejo, Miguel Ángel López-Manchado, Marianella Hernández Santana

**Affiliations:** Institute of Polymer Science and Technology (ICTP), Spanish National Research Council (CSIC), Juan de la Cierva 3, 28006 Madrid, Spain

**Keywords:** epoxidized natural rubber, polycaprolactone, thermoplastic elastomers (TPEs), alginic acid, alginates, self-healing materials

## Abstract

New bio-thermoplastic elastomer composites with self-healing capacities based on epoxidized natural rubber and polycaprolactone blends reinforced with alginates were developed. This group of salts act as natural reinforcing fillers, increasing the tensile strength of the unfilled rubber from 5.6 MPa to 11.5 MPa without affecting the elongation at break (~1000% strain). In addition, the presence of ionic interactions and hydrogen bonds between the components provides the material with a thermally assisted self-healing capacity, as it is able to restore its catastrophic damages and recover diverse mechanical properties up to ~100%. With the results of this research, an important and definitive step is planned toward the circularity of elastomeric materials.

## 1. Introduction

One of the agents that has a notable impact in environmental pollution is the uncontrolled management of polymer waste [1]. However, at the same time, polymers are a key player in achieving the Sustainable Development Goals (SDGs) of the United Nations (UN) [2]. There is no doubt about the importance of polymers for our development and for achieving true environmental sustainability, but not all polymers have the same impact on the ecosystem. Despite the bad press of the different types of polymers, plastics can be environmentally compatible because they can be reprocessed (with proper management). However, elastomers and thermosets cannot be due to their crosslinked chemical structure, which is responsible for their main characteristics as well as their limited flow [3].

Nevertheless, living without elastomers is not an option since their main characteristics are difficult to imitate. In the context of the circular economy [4], numerous strategies have been presented to reduce those consequences. Devulcanization is a widely studied option [5]. Another strategy is the development of thermoplastic elastomers (TPEs), which are formed by two different phases, one thermoplastic and another elastomeric, which means that the material can be melted and, thus, processed again. Other strategies are also framed within the concepts of the 7Rs of the circular economy: recover [6], reuse [7], redesign [8], reduce [9], renew [10], recycle [11], and repair [12].

The concept of self-healing (as a repair strategy) has gained special interest within the scientific community in the last 20 years [13,14,15,16,17,18]. Self-healing is understood as the ability of materials to repair or restore damage automatically, autonomously, or by applying an external stimulus [19]. In general, there are two approaches to achieve this feature: intrinsic self-healing, which is associated with the use of dynamic chemistry and supramolecular interactions, and extrinsic self-healing, in which an external healing agent is incorporated into the material and is responsible for sealing the damage. This agent is mainly confined in capsules or in vascular networks [19].

Repairability has mostly been studied in pure elastomeric materials, particularly those with intrinsic mechanisms [20,21]; however, in the field of self-healing TPEs there are only a few works [22,23,24,25] that show important improvement with favorable results. As an example, Lai et al. [24] prepared blends of natural rubber (NR) with polycaprolactone (PCL) combined with low amounts of acrylic acid (AA) in order to provide healing capabilities. The healing performance was tested on specimens with a 50% thickness cut after a 5 min thermal treatment at 60 °C or 80 °C. The self-healing efficiency was measured from the retention of tensile strength, and the best result was achieved for the samples heated at 80 °C due to the higher chain mobility at high temperatures (above the melting point of the PCL, ~70 °C).

In this work, all previous concepts have been used to develop new reinforced bio-TPEs with self-healing capacities through the use of additives that are mostly of natural origin, renewable, or biodegradable. This capacity has been achieved by combining intrinsic (such as hydrogen bonds and ionic interactions) and extrinsic self-healing mechanisms (from the flow of a dispersed thermoplastic inside the elastomer). For this, epoxidized natural rubber (ENR) was selected as the elastomeric matrix, and PCL was selected as the thermoplastic phase. In addition, to increase the sustainability of the prepared materials, the use of conventional reinforcing additives, such as CB and silica, was replaced by different natural fillers, specifically alginic acid salts or alginates, for the first time in a proper vulcanized material. All the prepared TPEs were thoroughly characterized by studying their morphologies and rheometric, thermal, and mechanical properties.

## 2. Materials and Methods

### 2.1. Materials and Compounding

Epoxidized natural rubber (ENR) with 25 mol% epoxidation (ENR 25) was supplied by the Tun Abdul Razak Research Centre (TARRC) of the Malaysian Rubber Board (Hertford, United Kingdom) and was used as an elastomeric phase. PCL (in the form of pellets, *M_n_* = 80,000) for use as a thermoplastic phase and dicumyl peroxide (DCP) for use as a as crosslinking agent (with a 1.56 g/mL density and a melting point of 110 °C) were purchased from Merck (Madrid, Spain). Zinc oxide (ZnO) from Scharlau (Barcelona, Spain) was also used.

**Figure 1:**
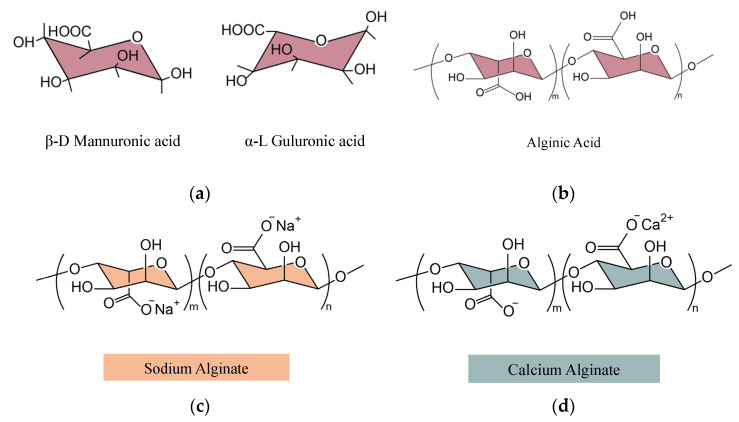
Chemical structure of (**a**) alginic acid G and M blocks, (**b**) A, (**c**) Na-A, and (**d**) Ca-A.

Alginic acid and alginates were selected as biofillers due to their renewable origin. Alginic acid (A) is a polysaccharide obtained from the extracellular matrix of Macrocystis pyrifera (a brown algae), with approximately 39% α-L-guluronic acid (G) blocks and 61% β-D-mannuronic acid (M) blocks (Figure 1a) linked with an o-glycosidic bond (with a molecular weight of ~240 kDa, Figure 1b). Two commercially available alginic acid salts (alginates) were also used: sodium alginate (Na-A) (Figure 1c) and calcium alginate (Ca-A) (Figure 1d). All these bioproducts were acquired from Merck (Madrid, Spain).

**Figure 2:**
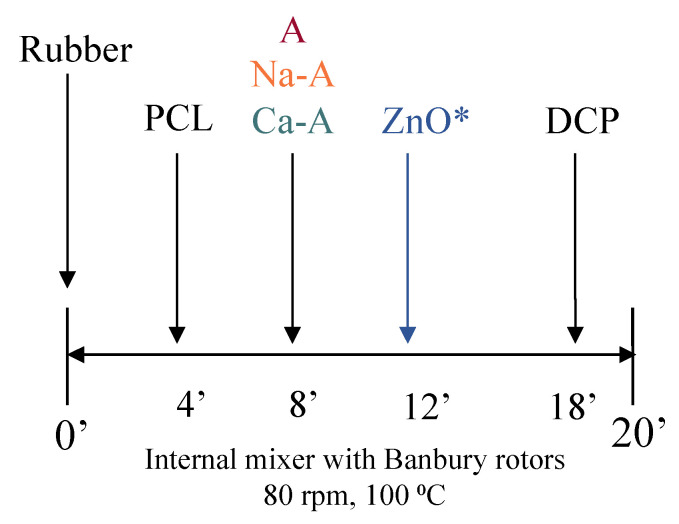
Scheme of the internal mixing process. (*) ZnO was only added in compounds with A.

The compounds were obtained after a regular rubber mixing procedure in a Haake Rheocord 9000 internal mixer (Thermo Fisher Scientific, Waltham, MA, USA) using Banbury rotors. The mixing was carried out at 100 °C with a rotor speed of 80 rpm for 20 min. The order of addition of the ingredients is shown in Figure 2. The recipes of the prepared compounds are presented in Section 3.

### 2.2. Vulcanization and Testing

#### 2.2.1. Rheometric Properties

An MDR 2000 Moving Die Rheometer (Alpha Technologies, formerly Monsanto, Heilbronn, Germany) was used to determine the rheometric properties in isothermal conditions. A ~4 g sample was placed between polyester films in an oscillating die with an oscillation arc of 0.5° and a 1.7 Hz frequency for 60 min. Temperature sweeps between 110 °C and 160 °C were used to obtain the optimal vulcanization temperature.

#### 2.2.2. Vulcanization

The vulcanization of the different compounds was carried out in a P 200 P hydraulic press (Collin, Maitenbeth, Germany). A pressure of 200 bar was set using the optimal temperature and times(*t*_90_) obtained from the curing curves. Vulcanized square sheets of 2 mm thickness were obtained after heating and subsequently cooling for 5 min. The samples were cut using a P-VS 3000 Universal Sample Cutter (MonTech, Columbia City, IN, USA) that operates with an 8 kN force, with the necessary shapes and dimensions for the different experimental techniques.

#### 2.2.3. Crosslink Density

The crosslink density was determined as an indirect measure of a swelling test in toluene. Five samples of each compound with a square shape (1 cm × 1 cm) and a thickness of 2 mm were cut, and the weight in air of each sample was measured (*m*_1_). Later, the samples were put into vials with fresh toluene for 72 h. After this time, the weight of the swollen samples was measured again (*m*_2_). Finally, the samples were dried for 24 h and the weight in air was measured (*m*_3_). The crosslink density (*v*) was calculated using Equation (1):

(1) $$v=\frac{\rho_{r}}{2M_{c}}$$

The Flory–Rehner equation provides the ratio between the density of the rubber (*ρ_r_*) and the molecular weight between the crosslinks (*M_c_*), as shown in Equation (2):

(2) $$\frac{\rho_{r}}{M_{c}}=-\frac{\ln\left(1-V_{r}\right)+V_{r}+\chi V_{r}^{2}}{V_{o}\left(V_{r}^{1/3}-\frac{V_{r}}{2}\right)}$$

where *V_o_* is the molar volume of toluene (106.20 cm^3^/mol), *χ* is the Flory–Huggins interaction parameter between the rubber and the solvent (0.42 for NR-Toluene), and *V_r_* is the volume fraction of rubber, calculated following Equation (3):

(3) $$V_{r}=\frac{\frac{m_{3}}{\rho_{r}}-V_{f}}{\frac{m_{3}}{\rho_{r}}-V_{f}+\frac{m_{2}-m_{3}}{\rho_{s}}}$$

where *ρ_s_* is the density of toluene (0.87 g/cm^3^) and *V_f_* is the volume fraction of the filler in the compound (if any).

#### 2.2.4. Differential Scanning Calorimetry (DSC)

A DSC 214 Polyma differential scanning calorimeter (Netzsch, Selb, Germany) was used to determine the thermal properties of the compounds in a nitrogen atmosphere. Three sweeps were made: a first heating sweep (from room temperature to 100 °C), which erased the thermal history of the material; a second cooling sweep (from 100 °C to −100 °C); and, finally, the third sweep (from −100 °C to 200 °C).

#### 2.2.5. Fourier-Transform Infrared Spectroscopy (FT-IR)

A Spectrum Two spectrometer (PerkinElmer, Waltham, MA, USA) was used in attenuated total reflection (ATR) mode. The tests were carried out on vulcanized samples in a 400 cm^−1^ to 4000 cm^−1^ wavenumber range with a resolution of 4 cm^−1^ and co-adding four scans per spectrum.

#### 2.2.6. Thermogravimetric Analysis (TGA)

The thermal stability of the compounds was studied using TGA. The analysis was carried out using a TGA 2 thermal analyzer (Mettler Toledo, Greifensee, Switzerland). The samples were heated from room temperature to 600 °C (in a nitrogen atmosphere) and up to 800 °C (in an oxygen atmosphere) using a heating rate of 10 °C/min. The spectra were analyzed using TA Universal Analysis software.

#### 2.2.7. Scanning Electron Microscopy (SEM)

The morphology of the samples was studied by scanning electron microscopy (SEM). An XL30 scanning electron microscope (Phillips, Eindhoven, The Netherlands) with a tungsten filament and an accelerating voltage of 25 kV was used. The cryogenic fracture zone, previously sputter-coated with a gold alloy, was observed.

#### 2.2.8. Hardness

A hardness test was carried out in a Shore A durometer Digi test (Bareiss, Oberdischingen, Germany) following the UNE-ISO 7619-1:2011 standard. Samples of 6 mm thickness, obtained by stacking, were used.

#### 2.2.9. Tensile Test

The stress–strain curve of the compounds was obtained using a universal testing machine 4204 (Instron, Norwood, MA, United States of America) with a load cell of 500 N. Following the UNE-ISO 37:2013 standard, uniaxial tensile tests were carried out in dumbbell-shaped samples (type 3) using a crosshead speed of 200 mm/min and an initial gap between clamps of 35 mm. The tensile strength (TS), the elongation at break (EB) (as the values of the stress and the strain at the breaking point, respectively), and the stresses at 100% (M100), 300% (M300), and 500% (M500) elongation, called as “modulus”, were recorded.

#### 2.2.10. Self-Healing Protocol

**Figure 3:**
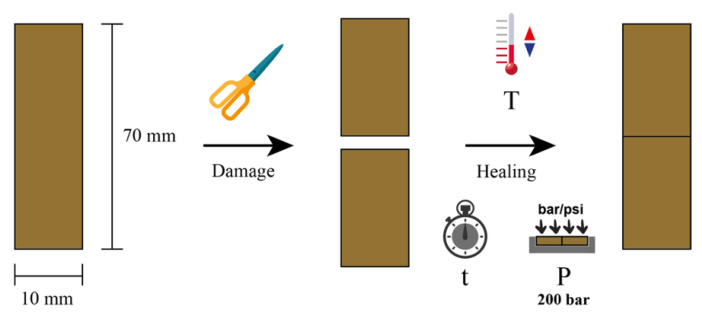
Scheme of the self-healing protocol.

The self-healing protocol involved two stages (Figure 3): first, the generation of damage in a rectangular specimen using a razor blade and, second, the repairability of this damage. For this, the two generated surfaces were manually placed in contact inside a mold with the dimensions of the pristine sample. Subsequently, different time (t) and temperature (T) conditions were applied under a pressure (P) of 200 bar. In this study, three temperatures (70 °C, 90 °C, and 110 °C) and three times (1 h, 5 h, and 12 h) were used. The study of different parameters allowed the optimization of the self-healing conditions.

The self-healing efficiency (η) of the compounds was measured as the retention of mechanical properties (M100, TS, and EB) after the repair of the macroscopic damage using Equation (4):

(4) $$\eta \left(\%\right)=\frac{P_{healed}}{P_{pristine}}\times 100$$

where *P_healed_* is the selected property of the healed sample and *P_pristine_* is the property of the pristine sample.

## 3. Results and Discussion

### 3.1. Novel TPEs Reinforced with Alginate

#### 3.1.1. Optimization of Processing Variables

The objective of this work was the preparation of biocomposites materials based on a TPE matrix reinforced with a natural filler known as alginate. The main motivation for the selection of this filler was its limited but potential use in rubber composites and its abundance in nature. Despite the advantages that this represents, it also brings some serious disadvantages related to processing, mainly because there are no standard conditions available in the literature that enable the optimal handling of these novel materials. Hence, the first stage of this work consisted of optimizing the process variables, specifically the vulcanization process. A constant ratio of 70/30 (100/42.86 in parts per hundred parts of rubber, phr) between ENR and PCL was selected, and two preliminary compounds, A0 (without fillers) and A10 (with 10 phr of A), were prepared (Table 1).

**Figure 4:**
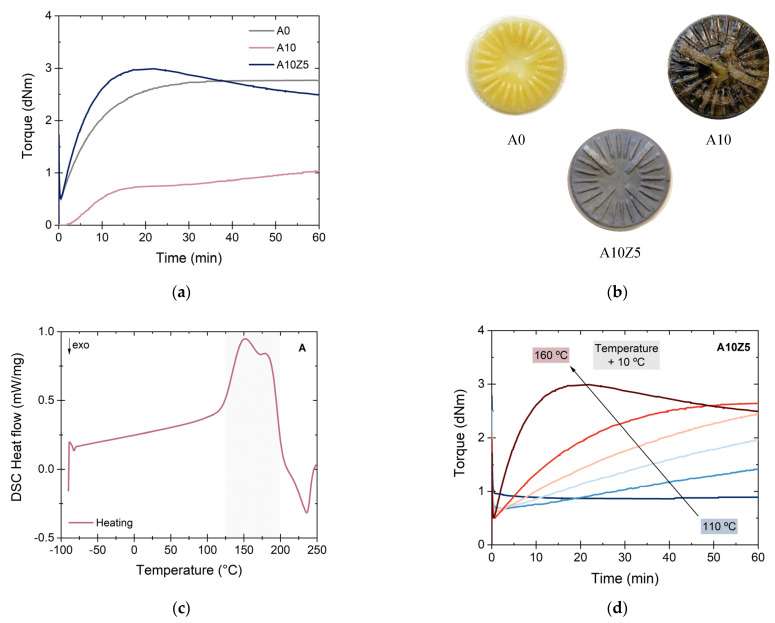
(**a**) Curing curves of preliminary compounds at 160 °C, (**b**) vulcanized rubber samples from the rheometer, (**c**) DSC thermogram of A, and (**d**) rheometric temperature sweep for A10Z5.

In order to find the vulcanization parameters, curing curves were obtained for both preliminary compounds (Figure 4a). A temperature of 160 °C was used as a standard value for most elastomeric materials. A drastic decrease in torque values was observed with the inclusion of 10 phr of A. This could be associated with a diluting effect of the filler in the TPEs or thermal instability at the selected temperature. In addition, a slowing of the vulcanization rate reaction was previously reported with the addition of acids or hindered phenols [26], which means that the chemical nature of the filler could be another reason for such a decrease. This dilemma was solved by observing the vulcanized parts obtained from the rheometric test. As can be observed in Figure 4b, the impeller of A10 presented a burnt, sticky, and foamed appearance. These characteristics indicate thermal instability at the test temperature.

To understand the thermal behavior of A, a DSC thermogram of the pure powder was performed (Figure 4c), showing two distinct zones: On one hand, an endothermic reaction between 120 °C and 200 °C was associated with the dehydration processes of the material [27,28]. Since alginic acid has a chemical structure composed of plenty of carboxylic and hydroxyl groups (Figure 1b), its tendency to absorb water is considerable and can affect its thermal performance. On the other hand, there was an exothermic reaction from 200 °C, which could be related to the degradation process of the material, but it will be studied more clearly by TGA.

From this, a strategy was proposed considering two points of view: first, the saturation of the free carboxylic groups in the alginic acid structure to decrease the water absorption rate and, second, a rheometric study at different temperatures to find the optimal value for the processing of this material. In this sense, a well-known method for the crosslinking of carboxylated elastomers was followed [8,29,30]. This family of rubbers can be vulcanized through metal oxides (such as ZnO, MgO, and CaO, among others) that, under the action of temperature, can form ionic interactions with the carboxylic groups, stabilizing them. For this purpose, ZnO, one of the most commonly used additives in rubber recipes, was selected in its conventional proportion (5 phr). The A10Z5 compound was designed following this strategy (see Table 1).

According to the results of the curing curves and the appearance of the vulcanized impeller at 160 °C (Figure 4b), it was possible to recover the typical behavior of a vulcanized rubber; however, the curve exhibited some “reversion”, an unconventional phenomenon in peroxide vulcanizates, which consists of a decrease in torque after reaching the maximum value (*M_H_*) [31]. This fact indicated the need to perform rheometric tests at different temperatures. Figure 4d shows the different curing curves of A10Z5 from 110 °C to 160 °C (with an increase of 10 °C per curve), and 150 °C was selected as the optimal temperature for the vulcanization of the TPEs due to its longer scorch time (always valuable for safe processing), lack of reversion, and *M_H_* values close to the vulcanization at 160 °C.

#### 3.1.2. Effects of the A and ZnO Contents

The contents of the new key ingredients in these TPEs were also optimized. Table 1 and Table 2 show the ratios used by varying the ZnO and A contents, respectively. For the ZnO variation, a constant content of 10 phr of A was selected (Table 1), while for the variation of A, a constant content of 5 phr of ZnO was selected (Table 2). This content is above the stoichiometric theoretical value required for the saturation of the available carboxylic groups (~3 phr). The use of an excess of ZnO would facilitate a higher degree of experimental saturation. Figure S1 shows the curing curves of these two sets of compounds.

In all cases, the increases in the *M_H_* and Δ*M* values confirmed the reinforcing character of A, reaching its maximum value at the highest amount (A10Z5, 10 phr). As for ZnO, two interesting effects should be considered: first, its character as a processing aid (it decreases the viscosity of the blend and, consequently, the value of the torques) and its role as a semi-reinforcing filler. It is clear that at contents below the saturation point there are decreases in the *M_H_* and Δ*M* values, even below the compound without ZnO (A10). However, these values increase from 3 phr of ZnO onwards, demonstrating an improvement in its reinforcing character by an enhanced effect between both components.

**Figure 5:**
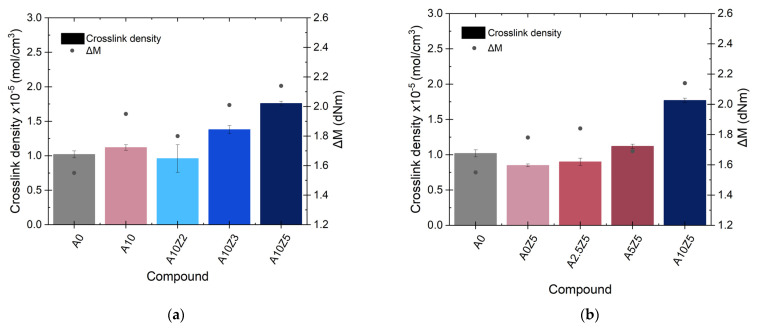
Relation between crosslink density and torques considering the (**a**) ZnO and (**b**) A contents.

The Δ*M* values can be correlated with the crosslink density of the material (Figure 5a,b), which is one of the key factors in the reinforcement of rubber vulcanizates [32]. This increasing trend can also be considered a validation of the strengthening effect of the combination of A with ZnO. Table S1 shows the compendium of the numerical values of all rheometric properties of this work.

**Figure 6:**
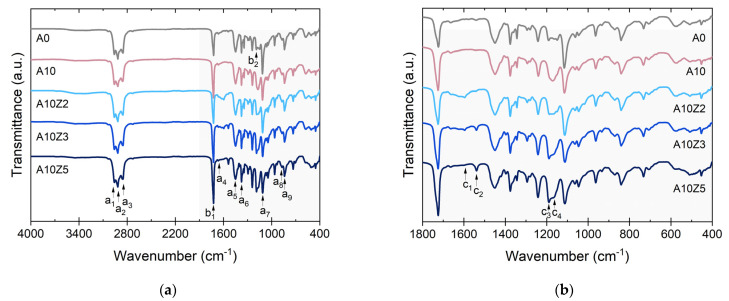
Infrared spectra of TPEs reinforced with A and ZnO: (**a**) general and (**b**) fingerprint zone.

The successful saturation of the carboxylic groups was followed by ATR-IR. Figure 6a shows the overall spectra of the compounds prepared by varying the ZnO content. The signals related to the chemical structures of the matrix were identified. For ENR, characteristic bands at 2960 cm^−1^ (a_1_), 2916 cm^−1^ (a_2_), and 2857 cm^−1^ (a_3_), associated with the stretching of the -CH bond; at 1660 cm^−1^ (a_4_) and 840 cm^−1^ (a_9_), related to the bending of the C=C bond; and at 1450 cm^−1^ (a_5_) and 1370 cm^−1^ (a_7_), corresponding to the bending of the -CH_2_ and -CH_3_ groups, were identified. Moreover, the epoxy ring was assigned at 1110 cm^−1^ (a_8_) and 870 cm^−1^ (a_8_).

Some of the bands of ENR are shared with the PCL, in addition to those of the proper thermoplastic structure such as the band at 1750 cm^−1^ (b_1_), distinctive of the stretching of the carbonyl group, C=O, and the band at 1190 cm^−1^ (b_2_), characteristic of C-O-C stretching [33]. In Figure 6b, the fingerprint of the compounds is presented. Four important bands were identified: the signals at 1597 cm^−1^ (c_1_) and 1540 cm^−1^ (c_2_), associated with the ionic coordination between the carboxylate ion (-COO) and the Zn^2+^ cations derived from ZnO [34], in addition to increases in the signals at 1190 cm^−1^ and 1165 cm^−1^, associated with the C-O-C stretching that has been reported as a consequence of the generation of secondary groups during ENR vulcanization with peroxides [12]

**Figure 7:**
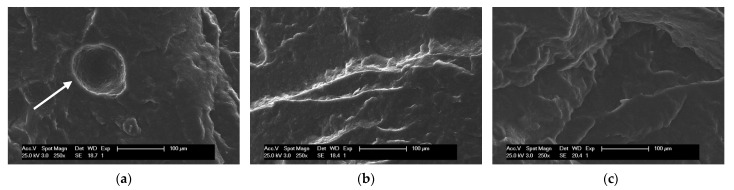
SEM photomicrograph of TPEs with A and ZnO: (**a**) A10Z2, (**b**) A10Z3, and (**c**) A10Z5. White arrow in (**a**) points to a space left by a bubble after vulcanization reaction.

A proof of the success of the saturation strategy was obtained from the observation of photomicrographs of the prepared compounds using SEM. Figure 7 shows photomicrographs at equivalent magnifications of the compounds A10Z2, A10Z3, and A10Z5. A homogeneous surface with well-defined fracture planes stands out, which would indicate good compatibility between the components of the TPE matrix. Additionally, no filler agglomerates were observed, which would indicate that good dispersion of A was achieved with the designed mixing protocol.

What is striking is the appearance of bubbles in A10Z2 (below the saturation point, Figure 7a, white arrow), which is further evidence of the dehydration that the unsaturated material undergoes at the process temperatures [27]. These bubbles disappeared in A10Z3 and A10Z5 (above the saturation point, Figure 7b,c), demonstrating the effect of saturation on the thermal behavior of the material in the first transition detected by DSC between 120 °C and 200 °C.

**Figure 8:**
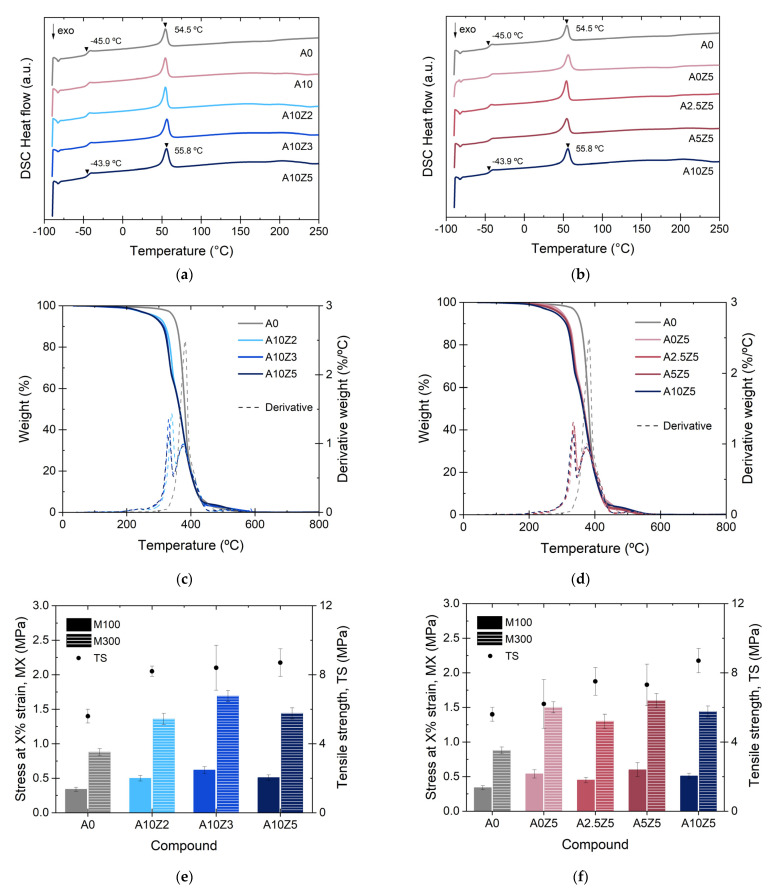
(**a**,**b**) DSC thermograms, (**c**,**d**) thermogravimetric curves, and (**e**,**f**) tensile properties of TPEs reinforced with A and ZnO.

To further investigate the thermal stability of the vulcanized compounds, DSC and TGA studies were carried out over a wide temperature range. Figure S2 shows the DSC curves of the matrix components, while Figure 8a,b show the DSC thermograms of the prepared composites. Two important transitions were detected. The first was the glass transition temperature, *T_g_*, between the matrix elements, which have individual values between −45 °C (ENR 25) and −60 °C (PCL). In the composites, the temperature value of the unfilled sample was detected at −45 °C (midpoint) and increased slightly to −43.9 °C. This effect was a result of the inclusion of the different additives, leading to an increase in the stiffness of the matrix, which hindered the mobility of polymeric chains. The second transition was an endothermic transition, the melting temperature, *T_m_*, associated with the melting of the PCL crystals. This value also slightly increased with the addition of A and ZnO as a result of a higher energy requirement that could be associated with the mobility constraints in the system.

The TGA results provide information on the second transition zone of A, above 200 °C, that was detected by DSC. Figure S2 shows the thermogravimetric curve of A, while Figure 8c,d show the weight change (%) and the first derivative as a function of the ZnO and A contents, respectively, of the prepared compounds. It was clearly observed that the composites present two important degradation episodes, which are associated with the presence of A (between ~200 °C and ~350 °C) [27,28], and the main degradation (around ~400 °C), associated with the matrix components. The incorporation of A affected the thermal stability of the compounds. Moreover, the neutralization reaction with ZnO had no positive effect on this process as it did in the dehydration reaction observed by DSC between 120 °C and 200 °C. In fact, a slight shift in the peak of the first derivative towards lower temperatures was observed with increasing ZnO contents. This shift was related to zinc-induced degradation of A. Zn^2+^ is an efficient Lewis acid that is able to coordinate hydroxyl groups and cleave to C-O bonds, particularly at high temperatures (>200 °C), accelerating the degradation of the filler [34].

However, it is important to note that these degradation reactions would be initiated at elevated temperatures compared to the ENR service conditions. This material never works at temperatures above 70 °C due to the phenomenon of rubber aging caused by environmental factors such as O_2_ and O_3_. Therefore, it can be stated that this weakening in thermal stability would have no effect on the typical applications of TPEs based on ENR and PCL.

#### 3.1.3. Mechanical Performance of TPEs Reinforced with A and ZnO

**Figure 9:**
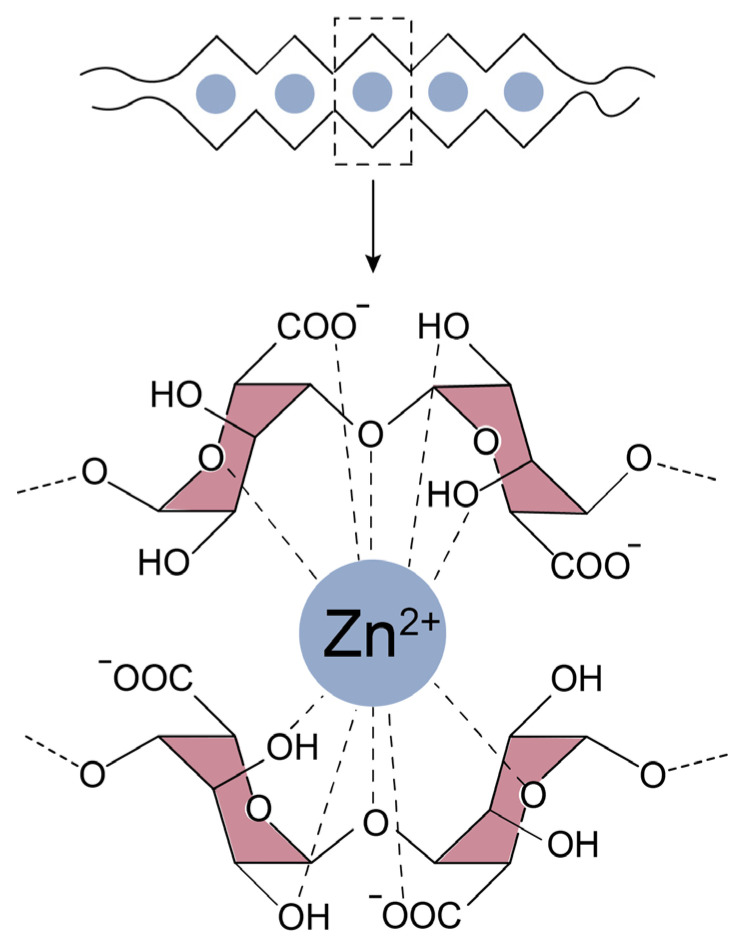
“Egg-box” model for A/ZnO combination.

Figure S3 shows the stress–strain curves as a function of the ZnO and A contents. In both cases, it is observed that the tensile curves exhibit typical elastomeric behavior. Figure 8e,f show the moduli values at 100% and 300% strains (M100 and M300) as well as the TS. For the moduli values, the trends are irregular. There seems to be a correlation with the A/ZnO ratio, but further tests should be performed to obtain a solid conclusion; however, it is undoubted that the TS increased consistently with the ZnO and A contents, which is further proof of the reinforcement effect achieved with the combination of these additives. This reinforcement effect, as mentioned above, may be the result of weak interactions such as hydrogen bonds with the elements of the TPEs and an ionic interaction between Zn^2+^ cations and the COO^−^ anion from A. According to the available literature, there is a widely accepted “egg-box” model (Figure 9) that explains the formation of an ordered coordination structure that “crosslinks” the alginate chains in the presence of divalent cations, providing stiffness, which could be acting as reinforcing sites [35]. This reinforcing character was also evidenced by the slight but steady increase in the hardness values with increasing ZnO and A. Table S2 shows the compendium of the numerical values of all mechanical properties of this work.

### 3.2. Effect of the Cation on the Reinforcement Effect of Alginates

The second part of this work consisted of the use of two new types of commercially available alginates based on sodium and calcium (Na-A and Ca-A) cations. The objective was to compare these salts with the in-house-synthesized zinc alginate (Zn-A) described in the previous section and derived from the combination of A and ZnO. It is important to highlight that Zn-A is not commercially offered. Table 3 summarizes the new TPEs recipes.

**Figure 10:**
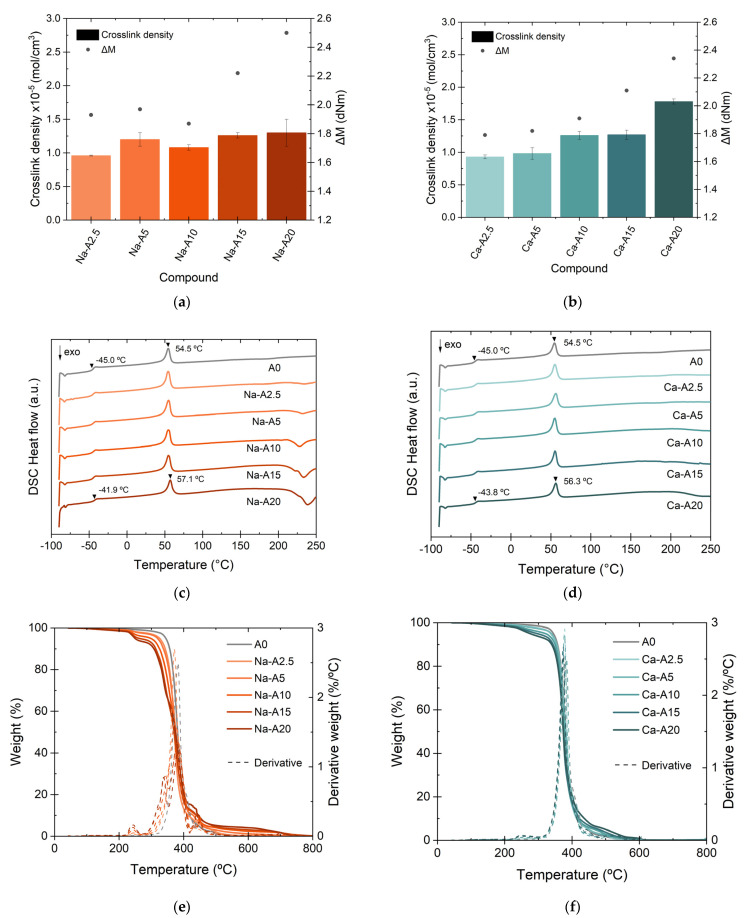
(**a**,**b**) Relation between crosslink density and torques, (**c**,**d**) DSC thermograms, and (**e**,**f**) thermogravimetric curves of Na-A- and Ca-A-reinforced TPEs, respectively.

Figure S1 and Table S1 show the curing curves and the rheometric property values of the composites with Na-A and Ca-A. The effect of the filler content on the rheometric properties of the blends is clearly observed. At low filler contents, very slight variations in the torque values are appreciated. However, the increase in the alginates content to values higher than 10 phr evidences an important rise in the *M_H_* value as well as in the vulcanization rate (the upward slope of the curves). These results indicate that these naturally occurring additives may require medium to high contents to achieve a good properties. This trend was also confirmed by the crosslink density values and Δ*M* (Figure 10a,b).

Interesting results were obtained regarding the thermal behavior of these materials. Figure 10c,d show the DSC thermograms of the Na-A and Ca-A composites, respectively. The increase in the *T_g_* of the matrix and the *T_m_* of the PCL with alginate content was again observed as a consequence of the reduced mobility upon the addition of fillers. However, the exothermic transition above 200 °C showed considerable intensity in the Na-A composites. This could indicate a higher thermal instability of Na-based ions.

**Figure 11:**
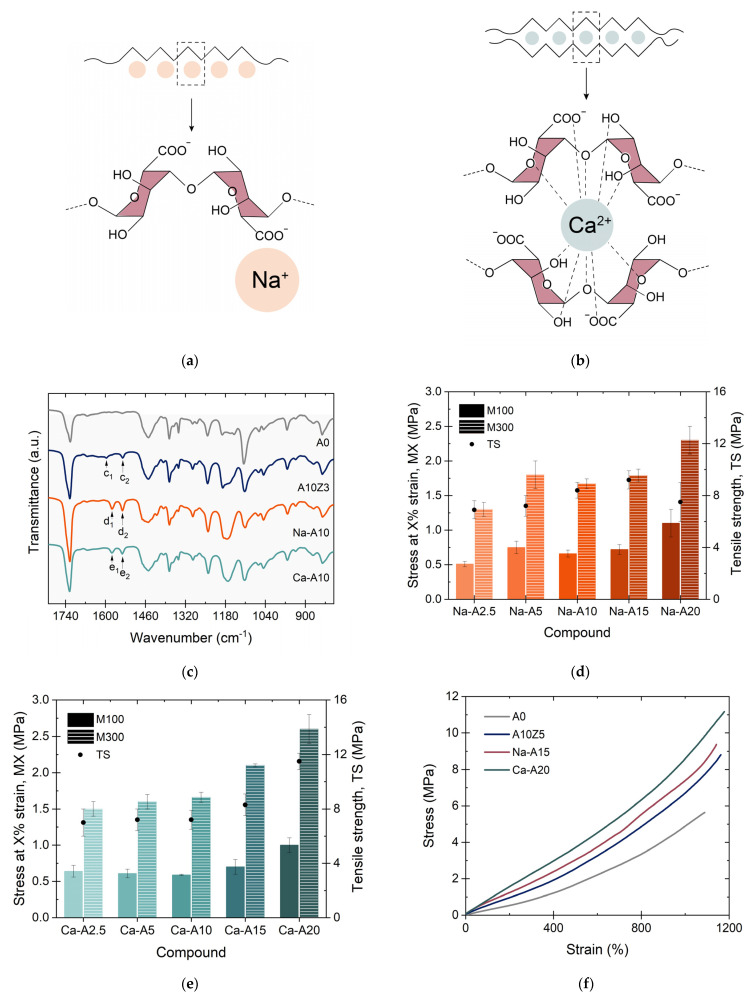
Model of the structures of (**a**) Na-A and (**b**) Ca-A. (**c**) Infrared spectra (ATR mode) of selected TPEs as a function of the cation involved, (**d**,**e**) tensile properties of Na-A- and Ca-A-based compounds, and (**f**) the best mechanical performance for each type of cation.

The TGA results allowed further investigation of the thermal stability of both ions. Figure 10e,f show the weight change (%) curves and the first derivative as a function of temperature. The high thermal stability of the Ca-A composites is clear, with only a slight shift towards lower temperatures in the main degradation, close to ~400 °C, in addition to other minor drops. The opposite case was obtained in the Na-A composites, whose thermal stability is seriously compromised with increasing alginate contents (as was also confirmed by DSC). This could be another sign of the thermal instability of the Na-based ions and could have its origin in the molecular structure proposed for these composites (Figure 11a,b). Due to its monovalent nature, Na^+^ is not able to form the “egg-box” structure achieved by divalent cations such as Zn^2+^ and Ca^2+^. Instead, a linear structure is obtained, with pendant groups and a lower impediment [36]. Furthermore, it is well-known that the intensity of the ionic interactions is directly proportional to the charge and inversely proportional to the distance between the ions (ionic radius); in this case, Ca^2+^ is able to form a more stable structure with a higher energetic requirement for its degradation, as confirmed by TGA.

The formation of these structures was confirmed by FT-IR. Three well-resolved composites with a constant content of 10 phr of A and its equivalent Na-based and Ca-based salts (A10Z3, Na-A10, and Ca-A10) were compared with the unfilled one (A0). In the spectrum, the appearance of two signals for each compound stood out (Figure 11c). The bands previously assigned to the systems with ZnO and A could also be identified in these compounds at slightly different wavenumbers around 1577 cm^−1^ and 1541 cm^−1^, which are associated with the ionic interactions between the carboxylate ion (COO^−^) and the Na^+^ and/or Ca^+2^ cations [36]. As mentioned above and has been reported extensively in the literature, these interactions are of a dynamic nature and will play a key role in the self-healing capacity of the TPEs.

#### Mechanical Performance of TPEs Reinforced with Na-A and Ca-A

The differences between the nature of the cation and the structures of alginates also carry over to the mechanical properties. Figure S3 and Table S2 show the stress–strain curves and the tensile property values of the TPEs, respectively.

All composites exhibited typical elastomeric behavior, as before, which was related to the dominance of the vulcanized rubber phase (in a higher proportion than the thermoplastic). Figure 11d,e summarize the values of M100 and M300 as well as TS for these sets of materials. It was generally observed that the composite with 20 phr of Ca-A (Ca-A20) reached the highest TS values among all composites prepared in this research (~11.2 MPa); on the other hand, the composites with Na-A exhibited a maximum at 15 phr of Na-A (Na-A15), which seems to be the limit that the material admits before losing its tensile properties.

On the other hand, unlike what happened with the ZnO-based composites, the moduli, particularly M300, exhibited a clear tendency to increase with the alginate content. This difference could be associated with the quality of the ionic interaction formed during mixing and vulcanization (A and ZnO) versus a more optimized commercial synthesis process (Na-A and Ca-A). Similar results were reported by Straccia et al. [37] in novel Zn-A hydrogels. However, in any case, the reinforcing effects of these two types of alginates were demonstrated.

The best vulcanizates of this group of blends also exhibited good compatibility between the matrix components, with a homogeneous surface and well-defined fracture planes, as in saturated composites with A (A10Z3 and A10Z5). Therefore, it could be concluded that the cation’s nature does not have a negative effect on the compatibility of the ENR and PCL or on the correct dispersion of the filler, as shown in the selected photomicrographs in Figure S4. All these facts, with the optimization of the processing variables, explain the outstanding mechanical performance achieved in the prepared composites.

### 3.3. Self-Healing Performance of Reinforced TPEs

For the study of self-healing capacity, it was mandatory to set a protocol of temperature, time, and pressure. For temperatures, the results of the thermal analysis carried out on the composites were considered. Temperature values above the melting of PCL (around ~60 °C) were selected to achieve the mobility of the chains and facilitate the flow towards the damage. Otherwise, the onset of dehydration reactions was observed at around 125 °C. Although in the fully vulcanized material the appearance of these reactions is almost imperceptible due to the difficulties of water absorption in this state, this value was selected as the upper limit. In this sense, the repair protocol was carried out in a hydraulic press at 200 bar pressure; at 70 °C, 90 °C, and 110 °C; for 1 h, 5 h, and 12 h. The choice of these times was based on preliminary work by the research group [19,38], while the pressure is the same as that used for the vulcanization of the TPEs.

Of all the prepared composites, the best of each cation series was selected in terms of their TS values: A10Z5, Na-A15, and Ca-A20, in addition to the unfilled composite, A0. The selection of TS as a comparative criterion was related to the required trade-off between this property and the self-healing capacity. It is well-known that both are antagonistic properties. The higher the TS, the lower the healing efficiency. Resolving this dichotomy remains a constant goal in the development of self-healing materials [39]. The stress–strain curves of the four study samples are shown in Figure 11f. The reinforcement characters of the different types of alginates were evidenced again since the TS value was improved from ~5.6 MPa in the unfilled compound to ~8.7 MPa (with 10 phr of A and 5 phr of ZnO), ~9.2 MPa (with 15 phr of Na-A), and ~11.5 MPa (with 20 phr of Ca-A), representing increases of up to ~55%, ~65%, and ~100%, respectively, without affecting the EB (~1000% strain).

**Figure 12:**
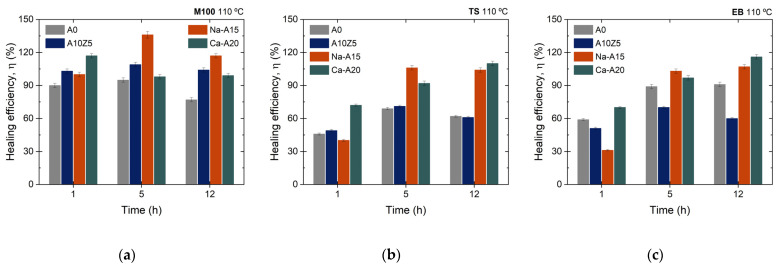
Self-healing efficiencies based on the recovery of (**a**) M100, (**b**) TS, and (**c**) EB at 110 °C for different times.

The 1 h healing protocol at different temperatures was used as a starting point. The results of the healing efficiency, measured as the recovery of the M100, TS, and EB values, are shown in Figure S5. For the M100 values, healing efficiencies higher than 70% were observed in all cases, which indicates the successful recovery of this functionality in the selected compounds. For TS, the results were diverse. At 70 °C, the best healing efficiency values (~66%) were obtained for the unfilled composite, A0. This result showed the basic influence of the extrinsic mechanism generated by the exclusive flow of the PCL in the TPEs. It is not strange that the filled composites presented lower efficiencies at this temperature due to the higher energetic requirements in hindered systems. When the temperature was increased up to 90 °C, equivalent efficiencies seemed to be reached for all reinforced composites (~50%), which were higher than the unfilled one (~46.3%). With this result, the effect of the higher mobility in combination with other intrinsic mechanisms began to be demonstrated (full details about the healing mechanism are explained in the next section). Finally, at 110 °C, the best efficiency was obtained for Ca-A20, with a recovery of ~72%. Thus, 110 °C was selected as the constant temperature for the time sweep. The results of the repair efficiency at 110 °C, during different times, are shown in Figure 12a–c.

Again, efficiencies higher than 70% for M100 were obtained for all developed composites, thus confirming the successful functional recovery of this property. As for the TS, the change was resounding. Optimal recovery values were reached in A0 (~62%), A10Z5 (~71%), and Na-A15 (>100%) in 5 h, while Ca-A20 (>100%) required 12 h. In this way, it was possible to obtain a successful recovery of TS in the composite with 10 phr of A and 5 phr of ZnO, in addition to a total recovery of this fundamental property with catastrophic damage in the composites with 15 phr of Na-A and 20 phr of Ca-A, which in turn exhibited total efficiencies (>100%) in their EB. These outstanding results represent a critical advance in the development of new self-healing and sustainable elastomeric materials.

#### Self-Healing Mechanism

**Figure 13:**
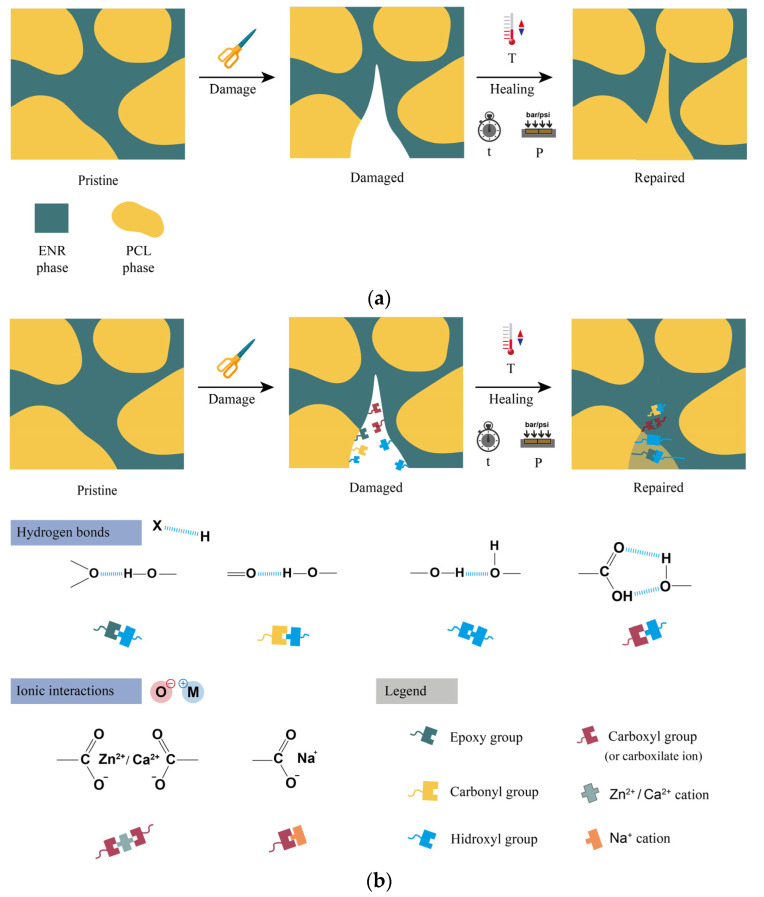
Scheme of self-healing: (**a**) extrinsic and (**b**) intrinsic mechanisms.

As previously stated, self-healing is achieved through two mechanisms, which are illustrated in Figure 13. First, an extrinsic mechanism related to the flow of the PCL. Due to its thermoplastic character, this polymer softens with increasing temperatures (above the *T_m_*), reaching absolute mobility of the polymeric chains and, therefore, flowing towards the damage zones, sealing the cracks. Second, two intrinsic mechanisms, such as the formation of reversible hydrogen bonds and ionic interactions. H-bonds can be formed between the free hydroxyl and carboxyl groups present in alginates with the epoxy groups in ENR 25, the carbonyl group of PCL, and intramolecularly with other similar free groups [40]. Meanwhile, according to the available evidence, the formation of ionic interactions between cations such as Zn^2+^, Na^+^, and Ca^2+^ and the carboxylate ion has a dynamic character [41]. This reversibility is activated with increasing temperature due to a mechanism known as “ion-hopping”, which consists of the cation’s migration towards an adjacent anion [42,43]; this ionic motion promotes mobility towards and in the damage zone, facilitating the repair of the material. Thus, this self-healing model involves the combination of three repair mechanisms (one extrinsic and two intrinsic). Some aspects require further analysis, but these outstanding preliminary results that allow the full recovery of mechanical properties open a new circular strategy in these exciting materials.

## 4. Conclusions

New bio-TPE composites were successfully designed using ENR/PCL, an elastomer of natural origin and a biodegradable thermoplastic, reinforced with alginic acid salts. An SEM analysis revealed both the good compatibility of the blend and the correct dispersion of the fillers. The incorporation of alginates provided certain thermal instability well above the regular service conditions of the ENR, while the reinforcing character was evidenced with tensile strength improvements of up to ~55%, ~65%, and ~100% for samples with 10 phr of A and 5 phr of ZnO, 15 phr of Na-A, and 20 phr of Ca-A, respectively, without affecting the elongation at break, which remained around ~1000% strain. From the point of view of self-healing, the presence of PCL facilitated an extrinsic healing mechanism determined by the flow of the thermoplastic through the rubber matrix at temperatures above its melting temperature. In addition, the incorporation of A, Na-A, and Ca-A promoted the formation of hydrogen bonds and ionic interactions, well-known intrinsic self-healing moieties. In the best performance, the repair efficiency of the unfilled material was increased from ~68% to ~100% with 20 phr of Ca-A and applying 110 °C for 12 h. All these composites have potential applications in common markets for TPEs, such as the automotive (exterior and interior parts, instrument panels, air ducts, and pipes), construction (extruded parts for doors and windows), industrial (shock absorbers), consumer (tool handles and covers), medical (valves and tubes), electronics (cables and mobile phone components), and sporting goods sectors. However, research must continue to ensure the final scalability of these materials, adjusting the recipes with the additional ingredients required for each type of application. Nevertheless, the promising results reported in this research represent a major contribution to the application of circular economy principles to rubbers and open an important path toward the development of new and more environmentally friendly materials.

## Figures and Tables

**Table 1:** Rubber recipes of TPEs reinforced with 10 phr of A and ZnO (in phr).

Ingredients	A0	A10	A10Z2	A10Z3	A10Z5
ENR 25	100	100	100	100	100
PCL	42.86	42.86	42.86	42.86	42.86
A	0	10	10	10	10
ZnO	0	0	2	3	5
DCP	0.8	0.8	0.8	0.8	0.8

**Table 2:** Rubber recipes of TPEs reinforced with different contents of A and 5 phr of ZnO (in phr).

Ingredients	A0Z5	A2.5Z5	A5Z5	A10Z5
ENR 25	100	100	100	100
PCL	42.86	42.86	42.86	42.86
A	0	2.5	5	10
ZnO	5	5	5	5
DCP	0.8	0.8	0.8	0.8

**Table 3:** Rubber recipes of TPEs reinforced with Na-A and Ca-A ^1^.

Ingredients	X2.5	X5	X10	X15	X20
ENR 25	100	100	100	100	100
PCL	42.86	42.86	42.86	42.86	42.86
Na-A/Ca-A (X)	2.5	5	10	15	20
DCP	0.8	0.8	0.8	0.8	0.8

^1^ In the designation of the compounds, “X” is replaced by Na-A or Ca-A depending on the filler used.

## Data Availability

The data that support the findings of this study are available on request from the corresponding author, M.H.S.

## Supplementary Materials

The following supporting information can be downloaded at: https://www.mdpi.com/article/10.3390/polym14214607/s1, Figure S1: Curing curves; Table S1: Rheometric property values; Figure S2: DSC curves for pure matrix components and thermogravimetric curve for A; Figure S3: Stress–strain curves; Table S2: Mechanical property values; Figure S4: SEM photomicrographs of selected Na-A and Ca-A TPEs; Figure S5: Temperature effect on self-healing efficiency.
