# Supplementary material for: Development of Sustainable, Mechanically Strong, and Self-Healing Bio-Thermoplastic Elastomers Reinforced with Alginates
Source: Polymers (Basel). 2022 Oct 30;14(21):4607. doi: 10.3390/polym14214607 (PMC9653809; doi:10.3390/polym14214607)

SUPPLEMENTARY MATERIAL

Sustainable, Mechanically Strong, and Self-Healing Bio-Based Thermoplastic Elastomers Reinforced with Alginates

Saul Utrera-Barrios, Ornella Ricciardi, Sergio González,
Raquel Verdejo, Miguel Ángel López-Manchado and Marianella Hernández Santana*

Institute of Polymer Science and Technology (ICTP-CSIC), Juan de la Cierva 3, 28005 Madrid, Spain.

* corresponding author: marherna@ictp.csic.es

Figure S1. Curing Curves.

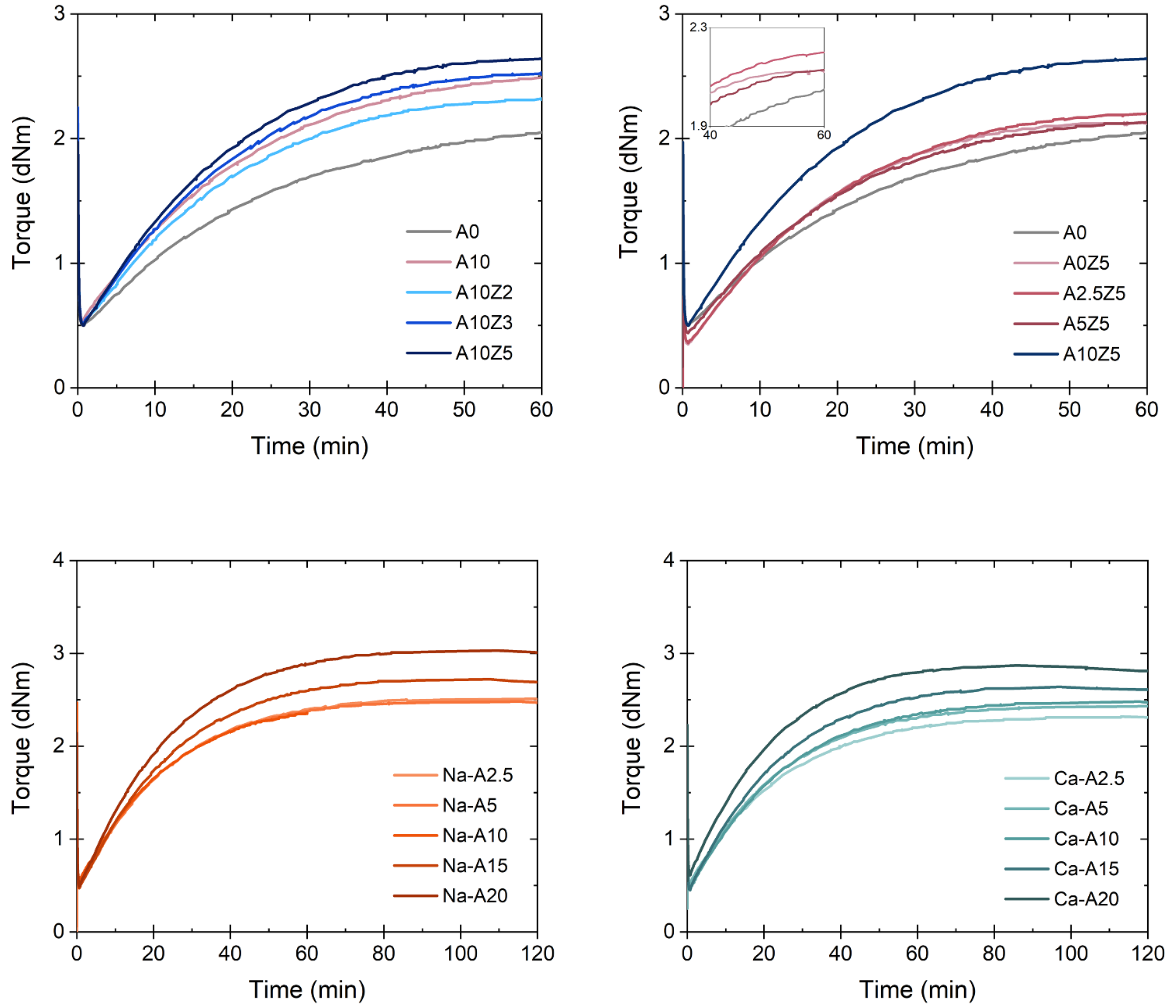

Table S1. Rheometric properties values.

Parameter	A0	A10	A10Z2	A10Z3	A10Z5
t_{s1} (min)	22.10	14.67	15.91	13.71	12.35
t_{90} (min)	42.74	39.26	36.38	36.85	35.68
M_L (dNm)	0.50	0.54	0.52	0.52	0.50
M_H (dNm)	2.05	2.49	2.32	2.53	2.64
ΔM (dNm)	1.55	1.95	1.80	2.01	2.14

Parameter	A0Z5	A2.5Z5	A5Z5	A10Z5
t_{s1} (min)	15.24	15.48	17.30	12.35
t_{90} (min)	34.42	37.09	37.87	35.68
M_L (dNm)	0.35	0.36	0.44	0.50
M_H (dNm)	2.13	2.20	2.13	2.64
ΔM (dNm)	1.78	1.84	1.69	2.14

Parameter	Na-A2.5	Na-A5	Na-A10	Na-A15	Na-A20
t_{s1} (min)	15.79	15.79	15.31	13.87	11.50
t_{90} (min)	41.35	42.48	42.25	42.39	41.59
M_L (dNm)	0.52	0.52	0.52	0.50	0.64
M_H (dNm)	2.45	2.49	2.39	2.72	3.14
ΔM (dNm)	1.93	1.97	1.87	2.22	2.50

Parameter	Ca-A2.5	Ca-A5	Ca-A10	Ca-A15	Ca-A20
t_{s1} (min)	18.01	17.74	17.31	15.16	12.33
t_{90} (min)	42.36	41.96	43.10	42.81	40.82
M_L (dNm)	0.55	0.50	0.47	0.48	0.59
M_H (dNm)	2.34	2.32	2.38	2.59	2.93
ΔM (dNm)	1.79	1.82	1.91	2.11	2.34

Figure S2. DSC curves for pure matrix components and thermogravimetric curve of A.

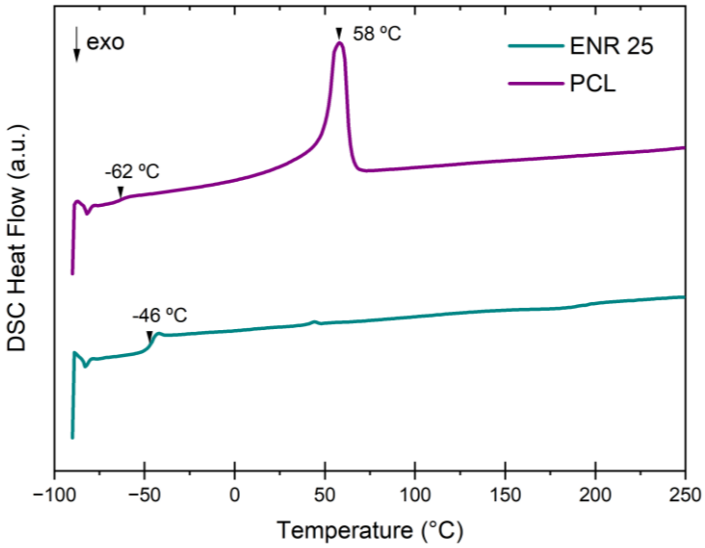

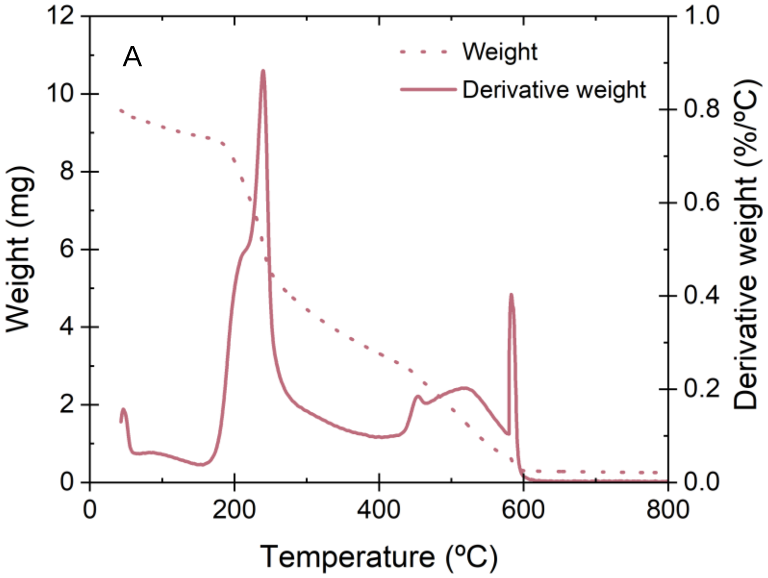

Figure S3. Stress-strain curves.

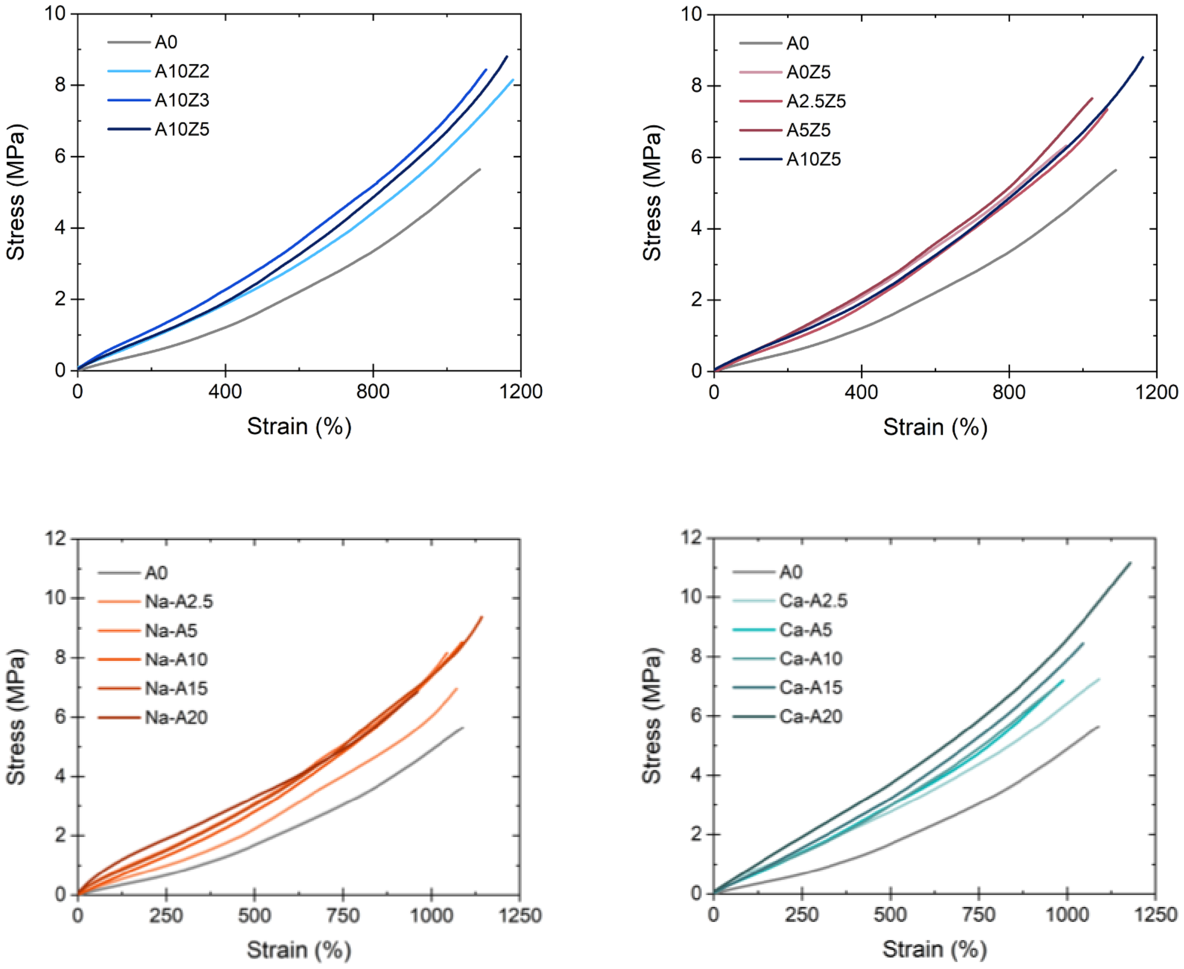

Table S2. Tensile properties values.

Parameter	A0	A10Z2	A10Z3	A10Z5
M100 (MPa)	0.34 ± 0.03	0.50 ± 0.04	0.62 ± 0.05	0.51 ± 0.04
M300 (MPa)	0.88 ± 0.05	1.36 ± 0.08	1.69 ± 0.08	1.44 ± 0.08
M500 (MPa)	1.66 ± 0.06	2.4 ± 0.1	2.9 ± 0.1	2.6 ± 0.1
TS (MPa)	5.6 ± 0.4	8.2 ± 0.3	8 ± 1	8.7 ± 0.8
EB (%)	1129 ± 64	1185 ± 23	1086 ± 243	1153 ± 72
Hardness (Shore A)	32 ± 5	40 ± 5	41 ± 5	42 ± 5

Parameter	A0Z5	A2.5Z5	A5Z5	A10Z5
M100 (MPa)	0.54 ± 0.06	0.45 ± 0.04	0.61 ± 0.08	0.51 ± 0.04
M300 (MPa)	1.50 ± 0.08	1.3 ± 0.1	1.6 ± 0.1	1.44 ± 0.08
M500 (MPa)	2.8 ± 0.1	2.6 ± 0.2	2.8 ± 0.1	2.6 ± 0.1
TS (MPa)	6 ± 1	7.5 ± 0.8	7 ± 1	8.7 ± 0.8
EB (%)	915 ± 118	1048 ± 81	1019 ± 98	1153 ± 72
Hardness (Shore A)	39 ± 5	39 ± 5	41 ± 5	42 ± 5

Parameter	Na-A2.5	Na-A5	Na-A10	Na-A15	Na-A20
M100 (MPa)	0.51 ± 0.04	0.75 ± 0.09	0.66 ± 0.05	0.72 ± 0.07	1.1± 0.2
M300 (MPa)	1.3 ± 0.1	1.8 ± 0.2	1.67 ± 0.07	1.79 ± 0.09	2.3 ± 0.2
M500 (MPa)	2.4 ± 0.1	3.0 ± 0.2	2.9 ± 0.1	3.0 ± 0.1	3.5 ± 0.2
TS (MPa)	6.9 ± 0.7	7.2 ± 0.8	8.4 ± 0.6	9.2 ± 0.7	7 ± 1
EB (%)	1039 ± 76	1013 ± 33	1079 ± 65	1156 ± 44	974 ± 134
Hardness (Shore A)	40 ± 5	43 ± 5	42 ± 5	43 ± 5	44 ± 5

Parameter	Ca-A2.5	Ca-A5	Ca-A10	Ca-A15	Ca-A20
M100 (MPa)	0.64 ± 0.08	0.61± 0.06	0.59 ± 0.01	0.7 ± 0.1	1.0 ± 0.1
M300 (MPa)	1.5 ± 0.1	1.6 ± 0.1	1.66 ± 0.07	2.1 ± 0.2	2.6 ± 0.2
M500 (MPa)	2.6 ± 0.1	2.8 ± 0.2	2.9 ± 0.1	3.4 ± 0.3	4.1 ± 0.2
TS (MPa)	7 ± 1	7.2 ± 0.8	7.2 ± 0.7	8.3 ± 0.8	11.5 ± 0.6
EB (%)	1115 ± 87	1028 ± 83	1000 ± 74	1035 ± 56,3	1150 ± 32
Hardness (Shore A)	41 ± 5	41 ± 5	40 ± 5	40 ± 5	46 ± 5

Figure S4. SEM photomicrograph of selected Na-A and Ca-A TPEs.

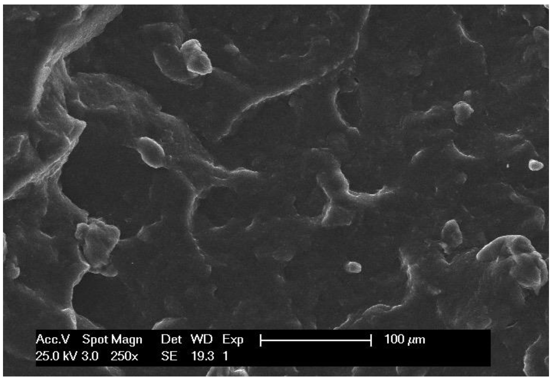

Na-A15

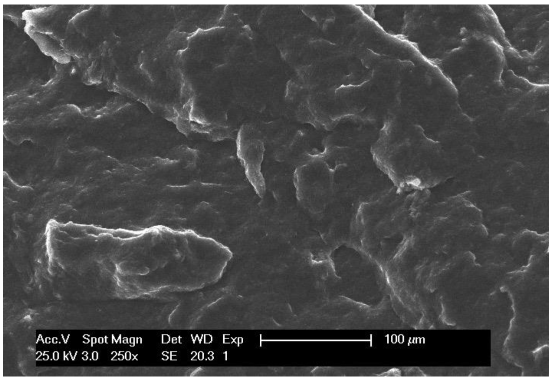

Ca-A20

Figure S5. Temperature effect on self-healing efficiency.

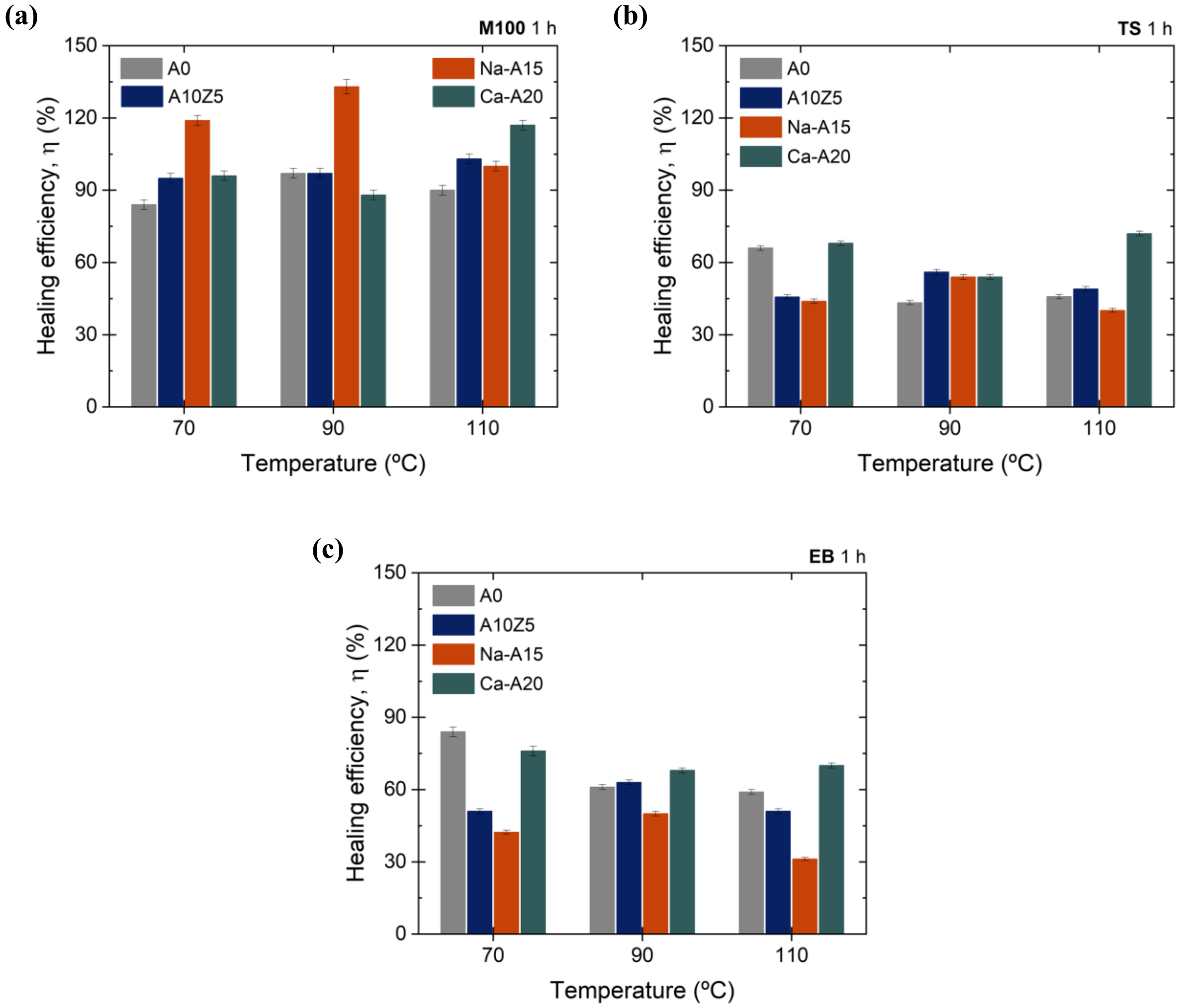

Supplement: Supplementary file 1 [file polymers-14-04607-s001.zip › polymers-1970432-supplementary.pdf]
